# Perceived Stress and Colorectal Cancer Incidence: The Japan Collaborative Cohort Study

**DOI:** 10.1038/srep40363

**Published:** 2017-01-16

**Authors:** Norimasa Kikuchi, Takeshi Nishiyama, Takayuki Sawada, Chaochen Wang, Yingsong Lin, Yoshiyuki Watanabe, Akiko Tamakoshi, Shogo Kikuchi

**Affiliations:** 1 Clinical Study Support, Inc., Nagoya, 460-0003, Japan; 2 Department of Public Health, Aichi Medical University School of Medicine, Nagakute, 480-1195, Japan; 3 Department of Epidemiology for Community Health and Medicine, Kyoto Prefectural University of Medicine Graduate School of Medical Science, Kyoto, 602-8566, Japan; 4 Department of Public Health, Hokkaido University Graduate School of Medicine, Sapporo, 060-8638, Japan

## Abstract

Colorectal cancer is the third most common cancer worldwide, and many risk factors for colorectal cancer have been established. However, it remains uncertain whether psychological stress contributes to the onset of colorectal cancer. Therefore, we conducted a large-scale prospective cohort study to confirm the association between perceived stress and colorectal cancer incidence. We identified 680 cases of colon cancer and 330 cases of rectal cancer during a maximum of 21-year follow-up of 61,563 Japanese men and women. Cox regression analysis adjusted for potential confounders revealed a significant association of perceived stress with rectal cancer incidence but not with colon cancer incidence. This finding is partly consistent with that from only one previous study that addressed an association between perceived stress and the risk of colorectal cancer. However, studies on this topic are sparse and warrant further exploration.

Colorectal cancer is the third most common cancer worldwide, accounting for 9.4% of all incident cancer in men and 10.1% in women^1^. Many risk factors for colorectal cancer, such as age, family history of colorectal cancer, physical inactivity, body mass index (BMI), cigarette smoking, heavy alcohol consumption, and nutritional practice have been established^1^,^2^,^3^,^4^. However, it remains uncertain whether psychological factors contribute to the onset of colorectal cancer, though many studies have examined the association of this cancer with various psychological factors, such as personality traits^5^, coping style^6^, depression^7^, and psychological stress including work stress^8^,^9^,^10^,^11^,^12^,^13^.

In the present study, we focused on psychological stress. Generally, psychological stress is measured by the occurrence of environmental events that are consensually judged as taxing one’s ability to cope, or by individual responses to such stressful life events^14^. Each type of stress measure may differentially reflect disease susceptibility^15^. To our knowledge, only one cohort study has assessed the association between psychological stress and colorectal cancer incidence^9^. In that study, there was no clear association between perceived stress and the risk of colon or rectal cancer. Regarding a study that addressed work stress, a large meta-analysis using pooled data from 12 cohort studies revealed no association of work stress with the risk of overall cancer or site-specific cancer, including colorectal cancer^8^. In contrast, the previous study that used the same cohort as the present study revealed a significant association between perceived stress and colon cancer mortality in women^16^. However, using colorectal cancer mortality as an outcome measure prevented distinguishing between risk and prognostic factors^9^. Therefore, we conducted this follow-up analysis to confirm the association between perceived stress and colorectal cancer incidence, instead of colorectal cancer mortality, using the final dataset of a large-scale prospective cohort study, the Japan Collaborative Cohort (JACC) Study^17^.

## Method

### Study population

The details of the study procedure were described elsewhere^17^. Briefly, 110,585 Japanese adults (46,395 men and 64,190 women) aged 40–79 years were enrolled between 1988 and 1990 in 45 locations throughout Japan. Most participants were recruited at the general health check-ups provided by municipalities.

The present study excluded 23 study areas where information on cancer incidence at follow-up or perceived stress at baseline was not available. Furthermore, 58 participants were excluded because they had a previous history of colorectal cancer. Another 3,421 participants were excluded because their responses regarding psychological stress were missing. Thus, a total of 61,563 participants (25,018 men and 36,545 women) were included in the present study.

Informed consent was obtained individually from each participant, except in a few study areas where informed consent was obtained at the group level after the purpose of the study and confidentiality of the data had been explained to community leaders. The JACC Study began before the ethical guidelines were first established by the Japanese government in 2002, and the Japanese ethical guidelines allows such established epidemiological studies to continue without obtaining additional personal informed consent^18^,^19^,^20^. The present study was approved by the ethics board at Nagoya University School of Medicine, where the central office of JACC Study was located^17^.

### Baseline measurement

At baseline, we used a self-administered questionnaire to obtain information about: age, body mass index (BMI) calculated as weight (kg)/height (m^2^), family history of colorectal cancer, smoking habit, alcohol drinking frequency, sleep duration per night, frequency of green leafy vegetables intake, walking time per day, bowel movements frequency, age of graduation (age at completion of full-time education), marital status, number of children, employment status.

For continuous variables, age and BMI were categorized into quartiles, age of graduation were categorized according to the education system in Japan, and sleep duration per night were categorized into three groups (~6.9, 7.0~7.9 or 8.0~ hours) because the distribution of sleep duration was concentrated in a narrow range from 7.0 to 8.0 hours. For categorical variables, sparse categories were combined together as shown in Table 1.

Perceived stress was assessed using the question “How much stress do you feel in your daily life?”, and responses were obtained using a four-point Likert scale: “little”, “moderate”, “high”, and “severe”. The last two categories were merged in the analysis because of the small number of incident colorectal cancer cases. The one-year test-retest reliability of this question was fair to good^21^ (weighted *κ* = 0.55)^22^, which is similar to that reported in the INTERHEART study (weighted *κ* = 0.53)^23^.

### Outcome ascertainment

Follow-up was conducted from enrollment until the end of 2009 in all areas, except three areas where follow-up was terminated at the end of 1999. During this period, population registries in the municipalities were used to ascertain the residential and vital status of the participants. In Japan, registration of death is required by the Family Registration Law, and theoretically provides complete mortality data. The cause and date of death of the participants were annually or biannually confirmed by reviewing death certificates in each area. The date of migration from the study areas was also annually or biannually verified by the investigators in cooperation with the local governmental office.

We ascertained the incidence of cancer by linking with records of population-based cancer registries, supplemented by a systematic review of death certificates. In some study areas, medical records from local hospitals were also reviewed^24^. The cancer incidence data were coded according to the 10th Revision of the International Statistical Classification of Diseases and Related Health Problems (ICD-10). We defined colon cancer as C18 and rectal cancer as C20 according to ICD-10.

### Statistical analysis

Although colon and rectal cancers are often treated together, several differences were identified between them^25^. Therefore, we evaluated each cancer type separately by sex for all analyses presented below.

Baseline participant characteristics were summarized by percentage for categorical variables and by mean and standard deviation (SD) for continuous variables separately by sex Spearman’s correlation coefficients and the 95% confidence intervals (CIs) between perceived stress and categorical variables were estimated to represent how they were correlated.

The person-years of follow-up were calculated from the baseline to the following events: diagnosis of colorectal cancer, death from any causes, migration outside the study areas, or the end of follow-up (the latter three events were treated as censored). The hazard rations (HRs) with the 95% CIs for colon or rectal cancer incidence were estimated using Cox proportional hazard regression models. For the analyses, we employed the same three models as in the previous study that used the first 7–9 years follow-up data of the same cohort^16^. The first model (age-adjusted) included only age as an independent variable. The second model (multivariate 1) added the following possible confounders to the first model: BMI, family history of colorectal cancer, smoking habit, alcohol drinking frequency, sleep duration per night, frequency of green leafy vegetables intake, walking time per day and bowel movement frequency, because these factors are known to influence colorectal cancer risk^1^. The third model (multivariate 2) added the following to the second model: age of graduation, marital status, the number of children and employment status. In the Cox regression analysis, missing values were treated as an additional category in each covariate. The percentages of the missing category in each covariate were shown in Table 1. There were no missing values in age, sex, and family history of colorectal cancers.

The proportional hazards assumption was checked by visual inspection of the log-log Kaplan-Meier curves. All tests were two-sided, and all analyses were performed using the SAS version 9.3 (SAS institute Inc., Cary, NC, USA).

## Results

Table 1 presents the baseline characteristics of the study population, stratified by sex. Regardless of sex, participants with higher perceived stress were, overall, likely to smoke more, drink alcohol more frequently, have higher education, be married, have more children, and work longer, as shown in a significantly positive Spearman’s correlation coefficient between perceived stress and each categorical variable. In contrast, participants with lower perceived stress were likely to be older, sleep longer, and eat green leafy vegetables more frequently, as shown in a significantly negative Spearman’s correlation coefficient. No clear patterns of correlation with perceived stress were observed in family history of colorectal cancer and walking time per day. Both variables were not significantly correlated with perceived stress. Sex differences were observed in the other variables: BMI and bowel movement frequency. These two variables showed significantly negative correlation with perceived stress in women, but not in men.

During the follow-up of 19–21 years (rectal cancer: mean of 13.0 years and 802,459 person-years; colon cancer: mean of 13.0 years and 800,588 person-years), 330 cases (220 men and 110 women) of incident rectal cancer, and 680 cases (356 men and 324 women) of incident colon cancer were ascertained.

Table 2 presents the HRs for rectal and colon cancer incidence associated with perceived stress at baseline, using three different models. Cox regression analysis adjusted for multiple potential confounders revealed a significant positive association of perceived stress with rectal cancer incidence in both men and women. In contrast, the results for colon cancer incidence revealed no statistically significant association with perceived stress. Although nominally significant associations were observed in the age-adjusted model in men, these nominal associations disappeared after adjusting for the other potential confounding variables.

## Discussion

Although the number of rectal cancer cases was small (*n* = 330) among 61,563 men and women followed up for a maximum of 19–21 years, perceived stress was significantly associated with the risk of rectal cancer, but not with the risk of colon cancer. The association between perceived stress and rectal cancer incidence remained consistent across the three statistical models, suggesting that our results are robust.

In a previous study that used the first 7–9 years follow-up data of the same cohort^6^, perceived stress was marginally associated with increased colon cancer mortality only in women (HR: 1.63, 95%CI: 1.00–2.64)^16^. However, using colorectal cancer mortality as an outcome measure in the study did not allow for a distinction between risk and prognostic factors^9^. Therefore, the present study employed colorectal cancer incidence as an outcome measure using another 12 years follow-up data and assured that the observed association is attributed to perceived stress as a risk factor, not as a prognostic factor. The difference between the previous and present studies is that our study identified an association of perceived stress with rectal cancer incidence in both men and women, but the former study identified a marginally significant association with colon cancer mortality only in women. Ultimately, all of these differences might come from the use of different outcomes, that is, the incidence and mortality of colorectal cancer.

To our knowledge, there is only one cohort study that has examined the effect of perceived stress on colorectal cancer incidence^9^. This cohort study found an association between either stress *intensity* or stress *frequency* and the risk of colorectal cancer, but failed to reveal consistent pattern of an association between perceived stress and the risk of colorectal cancer. In terms of stress *intensity*, a high level of perceived stress was significantly associated with the risk of rectal cancer only in men (HR: 2.94, 95%CI: 1.09–7.93), but no other significant associations were found for colon or rectal cancer in women. On the contrary, in terms of stress *frequency*, daily stress was significantly associated with the risk of colon cancer only in women (HR: 0.23, 95%CI: 0.07–0.73), but no other significant associations were found for colon or rectal cancer in men. It is quite difficult to interpret the whole picture from these findings because different stress measures (*intensity* or *frequency*) produced the opposite directions of effect (increase or decrease) on different cancer types (rectal or colon cancer) in different sex (men or women). However, the former finding is concordant with the present study finding that stress *intensity* was significantly associated with rectal cancer incidence. The differences between this and present cohort studies might be due to residual confounding in the previous one^9^, which did not adjust for several established risk factors for colorectal cancer, such as a family history of colorectal cancer and diet. Note that confounding can underestimate an association, that is, produce false-negative results^26^. Another source of discrepancies might have been regional differences between the two study areas, Denmark and Japan. For example, the incidence of colorectal cancer is stabilizing in western Europe, while the incidence is increasing rapidly in Japan^1^, and some of these geographic differences appear to be attributable to differences in dietary practices^27^,^28^.

As for work stress, a large meta-analysis using pooled data from 12 cohort studies revealed no association with overall cancer risk or site-specific cancer risk, including colorectal cancer risk^8^. However, work and non-work stress interact with each other and can have an impact on health^29^. Thus, we cannot adequately address the effect of psychological stress on colorectal cancer incidence based on the study only examining work stress.

On the other hand, there are several case-control studies on this topic^10^,^11^,^12^,^13^,^30^, although case-control studies are susceptible to biases. For example, cancer patients recall more stressful events than controls^31^, which could lead to a spurious association between psychological stress and the risk of colorectal cancer. Therefore, a type of prospective study design has been employed in which exposure information is obtained prior to knowledge of colorectal cancer diagnosis. As such, there is a study that examined the association between bereavement and cancer incidence^13^. That study identified 76 and 11,602 cases of incident colorectal cancer among 6,284 Jewish parents who lost one or two sons and among 1,019,255 individuals in the Jewish general population, respectively. The analysis revealed a significant association of bereavement with several site-specific cancers, but not with colorectal cancer, after adjusting for possible confounders. However, a small number of colorectal cancer cases among bereaved parents might result in a lack of statistical power, thus potentially increasing the likelihood of overlooking a true association, which is reflected by the wide 95% CIs of the odds ratios (ORs), *e.g.*, 0.85–3.09 among bereaved mothers of sons who died in accidents.

Overall, inconsistent results between previous studies and the present study might be attributable to low power or inadequate covariate adjustment in previous studies, the use of different stress measures, or regional differences among study areas. Nevertheless, interesting findings were consistently observed in the association pattern between psychological stress and cancer incidence. The highest HR for cancer incidence was observed at the moderate stress level, compared with HR at the lower and higher stress level. This reverse U-shaped association was observed in the former studies and the present study for colorectal cancer incidence, regardless of the presence or absence of significant association^9^. This association pattern was also observed in studies addressing other types of cancer such as breast and prostate cancer^32^,^33^,^34^. Because there was no simple dose-response relationship between stress and cancer incidence, confounding by an unknown factor would appear to be the most parsimonious explanation underlying the association between psychological stress and cancer incidence^33^. The finding of a reverse U-shaped association warrants further exploration because no clear explanation is apparent.

The present study had some limitations. We measured perceived stress only at baseline. This approach cannot address the influence of stress over time. A repeated measures design could uncover its influence on colorectal cancer incidence that would otherwise be missed. However, the fact that the influence of perceived stress on the risk of colorectal cancer was stronger during the first 9 years than during another 9 years of follow-up^9^ might alleviate this concern somewhat. Another measurement problem is the use of a single question to measure perceived stress in the present study. The single-item measure used in the present study showed fair to good test-retest reliability^22^ and satisfactory content, criterion, and construct validity^35^. However, compared with a multiple-item measure, a single-item measure is more influenced by random measurement error, which leads to a decrease in the power to detect a true effect^36^. Furthermore, for identification of colorectal cancer in the present study, population-based cancer registries were not available in four out of the 24 study areas; instead, medical records in local hospitals were reviewed in these four areas. The unsystematic identification of cancer cases in these areas could have caused a systematic error. Although the present study found significant association between perceived stress and rectal cancer incidence, 95% CI of the HR were still too wide (Table 2). This might be due to a lack of power from the relatively small number of cases of rectal cancer. Finally, we dealt with missing values in covariates by adding an extra category for missingness, which could potentially lead to biased results^37^. To assess this potential bias, we conducted a sensitivity analysis by excluding subjects with missing covariates, and found that the statistical significance of an association between perceived stress and rectal cancer incidence nearly vanished due to a lack of power from a decreased number of complete cases (Supplementary Table 1). Therefore, we could not rule out the potential bias from use of missing indicator variables in the present study.

## Conclusions

This large-scale prospective cohort study revealed a significant association of perceived stress at baseline with the risk of rectal cancer but not with the risk of colon cancer. However, wide 95% CI of the HR implicates the need for greater sample size. Studies on this topic are sparse and warrant further exploration.

## Additional Information

**How to cite this article:** Kikuchi, N. *et al*. Perceived Stress and Colorectal Cancer Incidence: The Japan Collaborative Cohort Study. *Sci. Rep.* **7**, 40363; doi: 10.1038/srep40363 (2017).

**Publisher's note:** Springer Nature remains neutral with regard to jurisdictional claims in published maps and institutional affiliations.

## Supplementary Material

Supplementary Table 1

## Figures and Tables

**Table 1:** Baseline characteristics of the participants stratified by sex.

Covariates: mean (SD)^a^		Men (*n* = 26,404)		Women (*n* = 3,8580)	
		%	Spearman’s *ρ* (95% CI)^b^	%	Spearman’s *ρ* (95% CI)^b^
Age (years) Men: 58.0 (10.3) Women: 58.3 (10.1)	~49	24.5	−0.24 (−0.25, −0.22)	22.8	−0.14 (−0.16, −0.13)
	50~57	22.6		23.5	
	58~65	28.4		27.8	
	66~	24.6		25.9	
	missing	0		0	
BMI (kg/m2) Men: 22.6 (3.0) Women: 22.9 (3.4)	~20.5	22.8	0.01 (−0.002, 0.02)	5.8	
	20.6~22.4	25.6		22.2	−0.03 (−0.004, −0.02)
	22.5~24.4	24.5		22.5	
	24.5~	21.9		25.7	
	missing	5.3		5.8	
Family history of colorectal cancer	no	97.8	0.00 (−0.01, 0.002)	97.5	−0.01 (−0.02, 0.001)
	yes	2.2		2.5	
	missing	0		0	
Smoking habit	never	19.6	0.02 (0.01, 0.03)	83.4	0.02 (0.01, 0.03)
	past	25.9		1.5	
	current	50.0		4.7	
	missing	4.5		10.5	
Alcohol drinking frequency (days/week)	0	24.3	0.02 (0.01, 0.03)	71.2	0.02 (0.01, 0.03)
	1~4	18.7		14.9	
	5~	46.6		5.0	
	missing	10.4		8.9	
Sleep duration/night (hours) Men: 7.5 (1.1), Women: 7.1 (1.1)	~6.9	16.7		27.8	
	7.0~7.9	32.1	−0.11 (−0.13, −0.10)	35.3	−0.12 (−0.13, −0.10)
	8.0~	47.0		31.4	
	missing	4.2		5.4	
Frequency of green leafy vegetables intake (times/week)	~1	10.5	−0.06 (−0.07, −0.04)	6.9	−0.05 (−0.06, −0.03)
	1~2	27.7		24.7	
	3~4	25.1		26.9	
	5~	26.0		30.7	
	missing	10.7		10.8	
Walking time/day (hours)	almost 0	10.0	−0.06 (−0.05, 0.08)	8.2	−0.02 (−0.03, 0.005)
	about 0.5	16.6		15.6	
	0.6~0.9	17.2		17.9	
	1~	40.1		41.2	
	missing	16.2		17.2	
Bowel movements frequency (times/day)	~0.9	10.6	−0.01 (−0.02, 0.01)	29.2	−0.04 (−0.05, −0.03)
	1	86.8		66.9	
	missing	2.7		3.9	
Age of graduation^c^ (years) Men: 17.1 (2.6) Women: 16.5 (2.1)	~14	8.5	0.13 (0.11, 0.14)	11.7	0.06 (0.05, 0.07)
	15~17	37.4		39.2	
	18~ 21	34.1		34.1	
	22~	6.2		1.1	
	missing	13.7		13.9	
Marital status	unmarried	5.6	0.02 (0.005, 0.03)	15.2	0.05 (0.04, 0.06)
	married	94.4		84.8	
	missing	0		0	
Number of children	0~1	6.7	0.06 (0.05, 0.08)	6.9	0.05 (0.04, 0.07)
	2	28.6		26.0	
	3	26.9		25.5	
	4~	12.0		15.0	
	missing	25.8		26.5	
Employment status	unemployed	15.7	0.16 (0.15, 0.18)	43.6	0.12 (0.11, 0.14)
	part-time	5.8		13.0	
	full-time	61.4		26.0	
	missing	17.1		17.4	

^a^Mean and standard deviation were shown only for continuously measured variables: age, BMI, sleep duration and age of graduation.

^b^Spearman’s correlation coefficient between perceived stress (mild, moderate, high and sever) and each categorized variable.

^c^Age at completion of full-time education.

**Table 2:** Hazard ratios for the incidence of colon or rectal cancer.

Perceived stress	Men			Women		
	Little	Moderate	High/Severe	Little	Moderate	High/Severe
Number at risk	3,973	13,507	5,609	6,120	20,622	7,118
Incidence of rectal cancer (*n*)	43	132	41	28	58	23
Person-years	52,159	181,869	78,227	79,421	271,339	93,274
Age-adjusted HR	Reference	2.31 (1.36–3.93)	1.99 (1.33–2.97)	Reference	2.83 (1.36–5.90)	1.74 (1.00–3.00)
Multivariate HR1^a^	Reference	2.29 (1.31–4.00)	1.91 (1.25–2.93)	Reference	3.65 (1.67–7.99)	2.07 (1.14–3.78)
Multivariate HR2^b^	Reference	2.16 (1.23–3.78)	1.75 (1.14–2.69)	Reference	3.20 (1.46–7.03)	1.83 (1.01–3.31)
Incidence of colon cancer (*n*)	77	194	79	60	191	65
Person-years	52,055	181,457	77,992	79259	270,549	93,152
Age-adjusted HR	Reference	1.62 (1.09–2.40)	1.37 (1.04–1.81)	Reference	1.14 (0.72–1.80)	1.06 (0.82–1.39)
Multivariate HR1^a^	Reference	1.31 (0.88–1.97)	1.10 (0.82–1.47)	Reference	1.06 (0.66–1.69)	0.99 (0.74–1.31)
Multivariate HR2^b^	Reference	1.21 (0.80–1.82)	1.05 (0.78–1.40)	Reference	1.01 (0.62–1.62)	0.94 (0.71–1.26)

^a^Adjusted for age, BMI, family history of colorectal cancer, smoking habit, alcohol drinking frequency, sleep duration per night, frequency of green leafy vegetables intake, walking time per day and bowel movement frequency.

^b^Adjusted for the covariates described above, as well as age of graduation, marital status, the number of children and employment status.
