# Supplementary material for: Perceived Stress and Colorectal Cancer Incidence: The Japan Collaborative Cohort Study
Source: Sci Rep. 2017 Jan 16;7:40363. doi: 10.1038/srep40363 (PMC5238416; doi:10.1038/srep40363)
Supplement: Supplementary Table 1 [file srep40363-s1.doc]

Scientific Reports

Perceived Stress and Colorectal Cancer Incidence: The Japan Collaborative Cohort Study

Norimasa Kikuchi^1,2^, Takeshi Nishiyama^2,*^, Takayuki Sawada^1,2^, Chaochen Wang^2^, Yingsong Lin^2^, Yoshiyuki Watanabe^3^, Akiko Tamakoshi^4^, Shogo Kikuchi^2,*^

1) Clinical Study Support, Inc., Nagoya, 460-0003, Japan

2) Department of Public Health, Aichi Medical University School of Medicine, Nagakute, 480-1195, Japan

3) Department of Epidemiology for Community Health and Medicine, Kyoto Prefectural University of Medicine Graduate School of Medical Science, Kyoto, Japan

4) Department of Public Health, Hokkaido University Graduate School of Medicine, Sapporo, 060-8638, Japan

Supplementary Table 1. Hazard ratios for the incidence of colon or rectal cancer based on a complete case analysis

|  |  | |  | Men | | |  | Women | | |
| --- | --- | --- | --- | --- | --- | --- | --- | --- | --- | --- |
|  | Perceived stress | |  | Little | Moderate | High/Severe |  | Little | Moderate | High/Severe |
| Multivariate  model1^a^ | Number at risk | |  | 2,667 | 10,373 | 4,468 |  | 3,959 | 14,758 | 5,148 |
|  | Incidence of rectal cancer (*n*) | |  | 25 | 89 | 30 |  | 12 | 35 | 16 |
|  | Person-years | |  | 33,910 | 139,933 | 62,146 |  | 50,109 | 197,607 | 67,190 |
|  |  | HR |  | Reference | 1.96 (1.00-3.86) | 1.68 (0.96-2.94) |  | Reference | 3.16 (1.18-8.46) | 1.47 (0.63-3.44) |
|  | Incidence of colon cancer (*n*) | |  | 50 | 150 | 59 |  | 32 | 128 | 44 |
|  | Person-years | |  | 33,829 | 139,548 | 61,995 |  | 49,974 | 197,059 | 67,072 |
|  |  | HR |  | Reference | 1.19 (0.74-1.90) | 1.10 (0.77-1.57) |  | Reference | 0.93 (0.54-1.62) | 0.81 (0.56-1.18) |
| Multivariate  model2^b^ | Number at risk | |  | 1,971 | 7,495 | 3,243 |  | 2,767 | 10,613 | 3,682 |
|  | Incidence of rectal cancer (*n*) | |  | 19 | 67 | 23 |  | 6 | 26 | 10 |
|  | Person-years | |  | 25,717 | 102,862 | 45,856 |  | 35,617 | 144,646 | 49,394 |
|  |  | HR |  | Reference | 1.42 (0.66-3.04) | 1.21 (0.66-2.23) |  | Reference | 3.52 (0.93-13.35) | 1.75 (0.53-5.75) |
|  | Incidence of colon cancer (*n*) | |  | 39 | 101 | 42 |  | 22 | 94 | 32 |
|  | Person-years | |  | 25,629 | 102,657 | 45,740 |  | 35590 | 144,280 | 49,284 |
|  |  | HR |  | Reference | 1.24 (0.69-2.22) | 1.14 (0.72-1.82) |  | Reference | 0.83 (0.43-1.59) | 0.71 (0.46-1.11) |

^a^Adjusted for age, BMI, family history of colorectal cancer, smoking habit, alcohol drinking frequency, sleep duration per night, frequency of green leafy vegetables intake, walking time per day and bowel movement frequency

^b^Adjusted for the covariates described above, as well as age of graduation, marital status, number of children, and employment status.
